# Associação do Genótipo e Fenótipo da Paraoxonase-1 com Angiografia Positiva para Doença Arterial Coronariana

**DOI:** 10.36660/abc.20210422

**Published:** 2022-08-24

**Authors:** Sara Saffar Soflaei, Mojtaba Baktashian, Kiana Hosseinpour Moghaddam, Maryam Saberi-Karimian, Negin Kosari, Seyed Mohammad Hashemi, Mohsen Mouhebati, Mahsa Amini, Mashallah Dehghani, Habibollah Esmaily, Mahmoud Ebrahimi, Homa Falsoleiman, Abolfazl Nosrati-Tirkani, Fatemeh Sadabadi, Gordon A. Ferns, Mansoor Salehi, Alireza Pasdar, Majid Ghayour-Mobarhan

**Affiliations:** 1 International UNESCO Center for Health-Related Basic Sciences and Human Nutrition Mashhad University of Medical Sciences Mashhad Irã International UNESCO Center for Health-Related Basic Sciences and Human Nutrition , Mashhad University of Medical Sciences , Mashhad – Irã; 2 Department of Biology Faculty of Sciences Ferdowsi University of Mashhad Mashhad Irã Department of Biology , Faculty of Sciences , Ferdowsi University of Mashhad , Mashhad – Irã; 3 Metabolic Syndrome Research Center Faculty of Medicine Mashhad University of Medical Sciences Mashhad Irã Metabolic Syndrome Research Center , Faculty of Medicine , Mashhad University of Medical Sciences , Mashhad – Irã; 4 Department of Cardiology School of Medicine Isfahan University of Medical Sciences Isfahan Irã Department of Cardiology , Chamran Hospital, School of Medicine , Isfahan University of Medical Sciences , Isfahan – Irã; 5 Cardiovascular Research Center School of Medicine Mashhad University of Medical Sciences Mashhad Irã Cardiovascular Research Center , School of Medicine , Mashhad University of Medical Sciences , Mashhad – Irã; 6 Department of Biostatistics and Epidemiology School of Health Mashhad University of Medical Sciences Mashhad Irã Department of Biostatistics and Epidemiology , School of Health , Mashhad University of Medical Sciences , Mashhad – Irã; 7 Biochemistry of Nutrition Research Center Faculty of Medicine Mashhad University of Medical Sciences Mashhad Irã Biochemistry of Nutrition Research Center , Faculty of Medicine , Mashhad University of Medical Sciences , Mashhad – Irã; 8 Brighton & Sussex Medical School Division of Medical Education Brighton Reino Unido Brighton & Sussex Medical School , Division of Medical Education , Falmer, Brighton – Reino Unido; 9 Department of genetics Faculty of medicine and genetics laboratory AL Zahra hospital Isfahan University of Medicine Isfahan Irã Department of genetics , Faculty of medicine and genetics laboratory AL Zahra hospital , Isfahan University of Medicine , Isfahan – Irã; 10 Division of Applied Medicine Medical School University of Aberdeen Aberdeen Reino Unido Division of Applied Medicine , Medical School , University of Aberdeen , Foresterhill, Aberdeen – Reino Unido

**Keywords:** Doença da Artéria Coronariana, Angiografia, Arildialquilfosfatase

## Abstract

**Fundamento:**

Tem sido demonstrado que um aumento dos níveis séricos de PON1 é protetor contra vários distúrbios. Foi relatado que vários polimorfismos de nucleotídeo único (SNPs, *single nucleotide polymorphisms* ) do gene PON1 estão associados a níveis e atividade de proteínas enzimáticas séricas.

**Objetivos:**

Investigar a associação de SNPs do PON1 e atividade da paraoxonase sérica com a doença arterial coronariana (DAC).

**Métodos:**

Foram estudados 601 pacientes não relacionados submetidos à angiografia coronária, incluindo aqueles com estenose >50% (N=266) e aqueles com estenose <30% (N=335). Os SNPs rs662 e rs840560 do gene da paraoxonase foram determinados utilizando o método ARMS-PCR e o SNP rs705379 foi genotipado utilizando análise de PCR-RFLP. A atividade da paraoxonase sérica foi medida utilizando paraoxon como substrato. O valor de p<0,05 foi considerado significante.

**Resultados:**

A atividade da paraoxonase sérica não foi significativamente diferente entre os grupos de estudo. Após ajuste para idade, sexo, hipertensão, diabetes mellitus e dislipidemia, o genótipo GG e o modelo codominante de rs662 foram positivamente associados a uma angiografia positiva (respectivamente, OR = 2,424, IC 95% [1,123-5,233], p <0,05, OR = 1,663, IC 95% [1,086-2,547]). A atividade da paraoxonase sérica foi significativamente maior no alelo G e variante GG do polimorfismo rs662, alelo A e variante AA de rs854560 e alelo C e variante CC de rs705379. A análise de haplótipos mostrou que o haplótipo ATC foi significativamente mais prevalente no grupo com angiografia negativa. A análise entre os grupos indicou que o alelo A de rs662 foi significativamente associado à menor atividade da paraoxonase no grupo com angiografia positiva (p=0,019).

**Conclusões:**

A presença do alelo G do polimorfismo de nucleotídeo único rs662 está independentemente associada ao aumento do risco de DAC.

## Introdução

A Organização Mundial da Saúde (OMS) relata que 71% das mortes a cada ano são devido a doenças não transmissíveis, das quais, 43% são devido à doença arterial coronariana (DAC).^1^ Isso também foi relatado como sendo de aproximadamente 46% no Irã em 2019,^2^ onde a prevalência de DAC está aumentando.^3 , 4^

O estresse oxidativo tem um papel fundamental no início e progressão da aterosclerose,^5^ bem como na patogênese da DAC e seus desfechos relacionados.^6^ Tem sido demonstrado que a lipoproteína de alta densidade (HDL, *high-density lipoprotein* ) possui propriedades antioxidantes e anti-inflamatórias nas quais a paraoxonase 1 (PON1) pode desempenhar um papel, diminuindo a produção de lipoproteína de baixa densidade (LDL, *low-density lipoprotein* ) oxidada durante o processo de peroxidação lipídica. A PON1 é uma esterase cálcio-dependente, cuja concentração sérica difere por etnia e geograficamente. Foi demonstrado que a diminuição da atividade de PON1 está associada a condições em que há estresse oxidativo, incluindo síndrome metabólica, DAC, Alzheimer e envelhecimento, e nesse caso, níveis aumentados de PON1 podem ser protetores.^7^ O *cluster* de genes da paraoxonase codifica três membros distintos, PON1-PON2 e PON3, localizados no cromossomo 7q21.3. Mais de 160 polimorfismos de nucleotídeo único (SNPs, *single nucleotide polymorphisms* ) foram identificados para o gene PON1,^8^ dos quais rs662, rs854560, rs705379 são descritos como associados aos níveis e atividade de proteínas enzimáticas séricas. Os SNPs rs662 e rs850560 estão localizados dentro de regiões codificadoras e resultam em uma substituição de aminoácidos^9 , 10^ enquanto o terceiro polimorfismo, rs705379, está localizado na região promotora.^11^ A presença do rs662 resulta em uma substituição de glutamina por arginina, aumenta a taxa de hidrólise de paraoxon e clorpirifós-oxon. Enquanto o polimorfismo rs850560, uma substituição do aminoácido leucina para metionina, também está associado à diminuição da PON^12^ sérica. O SNP rs705379 ocorre no local de ligação do fator de transcrição Sp1, e tem o maior efeito sobre a expressão de PON.^11^ Esse polimorfismo é responsável por aproximadamente 30% das variações nos níveis plasmáticos de PON1. O alelo C de rs705379 está associado a um aumento da atividade do promotor e, portanto, a expressão do gene PON1 é aumentada.^13^ Além disso, vários estudos mostraram que esse polimorfismo está associado a um risco aumentado de DAC, especialmente em jovens^14 - 16^ e em indivíduos com diabetes tipo 2.^17^

Como a DAC é uma doença importante em relação à mortalidade e os estudos mostram que existe uma associação entre PON1 e DAC, decidiu-se investigar essa associação na sociedade iraniana, principalmente no nordeste do país.^18^ Existem poucos estudos sobre a associação entre o genótipo ou fenótipo de PON1 e DAC no nordeste do Irã,^19 - 21^ e a atividade enzimática sérica não foi estudada juntamente com polimorfismos. O objetivo do presente estudo foi avaliar a associação entre polimorfismos de PON1 e atividade da paraoxonase com DAC em adultos iranianos que vivem no nordeste do país.

## Material e Métodos

### Desenho e população do estudo

Esse estudo de caso-controle foi realizado entre dezembro de 2014 e abril de 2017; foram recrutados 601 pacientes iranianos não relacionados que foram submetidos à angiografia coronária eletiva. Os pacientes foram encaminhados para angiografia devido a dor torácica ou sintomas equivalentes, como dispneia aos esforços. Com base nos resultados da angiografia, os pacientes foram divididos em dois grupos: aqueles com doença arterial coronariana obstrutiva com estenose coronariana >50% em pelo menos uma artéria coronária (N=266) (angiografia positiva) e pacientes com doença arterial coronariana não obstrutiva com estenose <30% nas artérias coronárias (N=335) (angiografia negativa).

Os dados demográficos incluindo sexo, idade, histórico de tabagismo, histórico pregresso de diabetes mellitus (DM), hipertensão (HAS) e dislipidemia foram coletados dos prontuários médicos. A pressão arterial sistólica (PAS) e a pressão arterial diastólica (PAD) foram medidas imediatamente antes do procedimento. Pacientes com doença autoimune, câncer ativo, trombofilia ou doença renal crônica foram excluídos.

As amostras de sangue foram coletadas antes do procedimento em tubos contendo EDTA para extração de DNA e em tubos sem anticoagulante para medida da atividade da paraoxonase. O soro foi separado por centrifugação do sangue por 15 min a 1000 rpm, que é velocidade recomendada pelo fabricante (BEHDAD, Irã) e armazenado a -80°C.

### Genotipagem

O DNA foi extraído do sangue com EDTA, utilizando um kit de isolamento de DNA genômico (Genet bio, Coréia) com base nas instruções do fabricante. A pureza e a quantificação do DNA foram determinadas por espectrofotometria UV (Infinite 200PRONanoQuant, Tecan).

Três SNPs dos genes PON1 foram genotipados. Utilizamos o método duplo ARMS-PCR para os SNPs rs662 e rs854560 e o método PCR-RFLP para o SNP rs705379. Detalhes sobre os primers utilizados e as condições de PCR são detalhados no Suplemento 1. A eletroforese em gel foi realizada utilizando agarose 2% com tampão Tris-Borato-EDTA (TBE) para os três SNPs. Para determinar os genótipos de rs705379, foram utilizadas 5 unidades de Bsh1236I (Thermo Scientific) por 16 horas a 37°C. O produto da PCR 109bp foi cortado em fragmentos de 67pb e 42pb e visualizado com Transiluminador UV. O sequenciamento foi realizado para confirmar a precisão das técnicas de genotipagem.

### Atividade da paraoxonase

A atividade da paraoxonase foi medida pela adição de 10 μL de soro a 290 μL de tampão Tris-HCl (100mmol/l, pH=8,0) contendo 1mmol/Lde CaCl_2_ e 1mmol/L de paraoxon (D9286, Sigma Chemical Company, Deisenhofen, Alemanha). A geração de p-nitrofenol foi medida a 405 nm e à temperatura ambiente, utilizando um leitor de placas (EPOCH, EUA) 3 e 6 minutos após a adição de paraoxon como substrato. A atividade da paraoxonase foi relatada em Unidades por litro de soro por minuto.

### Considerações éticas

Todos os participantes preencheram um formulário de consentimento informado por escrito. O Comitê de Ética da *Mashhad University of Medical Sciences* aprovou o protocolo do estudo (número de identificação: 930834).

### Análise estatística

Todos os dados foram analisados estaticamente utilizando o software *Statistical Package for Social Sciences* (SPSS Inc., IL, EUA). A normalidade dos dados foi verificada pelo teste de Kolmogorov-Smirnov. As variáveis com distribuição normal foram expressas em média ± desvio padrão (DP) e as variáveis sem distribuição normal foram descritas por mediana e intervalo interquartil. As variáveis categóricas foram expressas por número e porcentagem. Para a análise entre grupos, o teste de qui-quadrado foi usado para dados categóricos, e para dados quantitativos, utilizou-se o teste *t* para amostras independentes (para dados normalmente distribuídos) ou teste de Mann-Whitney e Kruskal-Wallis (para dados não distribuídos normalmente), respectivamente. Para indicar a associação entre SNPs e angiografia positiva, foram realizadas as análises univariada e multivariada com regressão logística binária, sendo expressas em OR (IC95%). A significância estatística foi estabelecida em p< 0,05. A análise de haplótipos foi realizada utilizando o SNPAlyze (versão demo, V8.1.1).

## Resultados

As diferenças nas características basais entre os grupos de estudo são mostradas na Tabela 1 . As diferenças entre a frequência dos três genótipos e a positividade da angiografia são mostradas na Tabela 2 . Após ajuste para idade, sexo, hipertensão, DM e dislipidemia, um modelo recessivo para o genótipo GG de rs662 foi significante entre as populações do estudo. Além disso, um resultado significante foi observado no modelo codominante para rs662.

**Tabela 1:** Características basais da população do estudo

Variável		Angiografia negativa (N=266)	Angiografia positiva (N=335)	p Valor
Idade (anos) (Média±DP)^1^		55,70±10,96	61,53±8,91	<0,001
Sexo (N%)^2^	*Masculino*	130 (48,9%)	227 (69,8%)	<0.001
	*Feminino*	136 (51,1%)	98 (30,2%)	
**Histórico de tabagismo (N %)^2^**		42 (16,0%)	52 (16,5%)	0,890
**Histórico de HTN (N %)^2^**		121 (45,5%)	192 (59,3%)	0,001
**Histórico de DM (N %)^2^**		70 (26,3%)	130 (40,5%)	<0,001
**Histórico de dislipidemia (N %)^2^**		93 (35,1%)	178 (55,3%)	<0,001
**IMC (N %)^2^**	*Normal (BMI<25)*	87 (37,5%)	104 (35,6%)	0.683
	*Sobrepeso (25≤BMI<30)*	101 (43,5%)	138 (47,3%)	
	*Obeso (BMI≥30)*	44 (19,0%)	50 (17,1%)	
**PAS (Média ± DP)^1^**		121,81±17,37	124,93±15,44	0,035
**PAD (Média ± DP)^1^**		76,12±10,42	77,75±8,75	0,063
**Atividade da paraoxonase sérica (U/L) (Mediana (IQR))^1^**		57,60(32,70-105,15)	52,20(30,38-95,18)	0,237

HTN: hipertensão; DM: diabetes mellitus; IMC: índice de massa corporal; PAS: pressão arterial sistólica; PAD: pressão arterial diastólica. ^1^ Análise por teste t ou teste de Mann-Whitney quando necessário. ^2^ Análise pelo teste do qui-quadrado.

**Tabela 2:** Associação entre polimorfismos de PON1 e DAC

Genótipos			Angiografia negativa (N=266)	Angiografia positiva (N=335)	Regressão univariada	Regressão multivariada^1^
rs662	*A*		0,70	0,68	1,093 (0,847-1,410)	1,062 (0,773-1,460)
	*G*		0,30	0,32		
	*AA*		112(44,8%)	150(48,1%)	Ref,	Ref,
	*AG*		121(48,4%)	121(38,8%)	0,747 (0,525-1,061)	0,685 (0,439-1,069)
	*GG*		17(6,8%)	41(13,1%)	1,913 (1,022-3,583)*	1,802 (0,827-3,925)
	*Modelo dominante*	*AA*	112(44,8%)	150(57,3%)	0,833 (0,632-1,233)	0,818 (0,535-1,250)
		*AG+GG*	138(55,2%)	162(51,9%)		
	*Modelo recessivo*	*AA+AG*	234(93,6%)	271(86,9%)	2,360 (1,274-4,373)**	2,424 (1,123-5,233)*
		*GG*	16(6,4%)	41(13,1%)		
	*Modelo aditivo*	*AA*	112(87,5%)	150(78,5%)	2,041 (1,076-3,871)*	2,080 (0,900-4,810)
		*GG*	16(12,5%)	41(21,5%)		
	*Modelo codominante*	*AG*	122(48,8%)	121(38,9%)	1,508 (1,077-2,113)*	1,663 (1,086-2,547)*
		*AA+GG*	128(51,2%)	190(61,1%)		
**rs854560**	*A*		0,62	0,67	0,944 (0,682-1,309)	0,836 (0,583-1,200)
	*T*		0,38	0,33		
	*AA*		88(35,5%)	131(42,3%)	Ref,	Ref,
	*AT*		131(52,8%)	149(48,1%)	1,053 (0,692-1,603)	0,918 (0,550-1,533)
	*TT*		29(11,7%)	30(9,7%)	0,299(0,694-1,383)	0,603(0,260-1,399)
	*Modelo dominante*	*AA*	86(34,7%)	130(41,9%)	1,004(0,643-1,568)	0,854(0,523-1,393)
		*AT+TT*	162(65,3%)	180(58,1%)		
	*Modelo recessivo*	*AA+AT*	220(88,7%)	279(90,0%)	0,770(0,381-1,556)	0,631(0,285-1,398)
		*TT*	28(11,3%)	31(10,0%)		
	*Modelo aditivo*	*AA*	86(75,4%)	130(81,2%)	0,789(0,374-1,665)	0,601(0,259-1,396)
		*TT*	28(24,6%)	30(18,8%)		
	*Modelo codominante*	*AT*	134(54,0%)	149(48,2%)	0,909(0,586-1,412)	0,990 (0,610-1,607)
		*AA+TT*	114(46,0%)	160(51,8%)		
**rs705379**	*C*		0,50	0,49	1,055 (0,798-1,394)	0,941(0,669-1,322)
	*T*		0,50	0,51		
	*CC*		49(24,1%)	68(21,9%)	Ref,	Ref,
	*CT*		109(53,7%)	171(55,0%)	0,941(0,574-1,544)	1,079(0,589-1,975)
	*TT*		45(22,2%)	72(23,2%)	1,098 (0,603-2,000)	0,585(0,418-1,761)
	*Modelo dominante*	*CC*	52 (25,6%)	67(21,4%)	1,086 (0,642-1,838)	1,143 (0,645-2,028)
		*CT+TT*	151(74,4%)	246(78,6%)		
	*Modelo recessivo*	*CC+CT*	159(78,3%)	242(77,3%)	1,001 (0,600-1,703)	0,820 0,460-1,462)
		*TT*	44(21,7%)	71(22,7%)		
	*Modelo aditivo*	*CC*	52(54,2%)	67(48,6%)	1,075 (0,561-2,063)	0,939 (0,458-1,924)
		*TT*	44(45,8%)	71(51,4%)		
	*Modelo codominante*	*CT*	107(52,7%)	175(55,9%)	0,952 (0,613-1,478)	0,791 (0,486-1,288)
		*CC+TT*	96(47,3%)	138(44,1%)		

A análise foi realizada usando regressão univariada e multivariada. * Valor p ≤ 0,05. **Valor p ≤ 0,01. ^1^ Ajustado para idade, sexo, hipertensão, diabetes mellitus, dislipidemia.

A análise do haplótipo mostrou que o haplótipo “ATC” apresentou diferença significante entre os dois grupos analisados (p=0,017) ( Tabela 3 ).

**Tabela 3:** Diferença entre frequências de haplótipos nos grupos de estudo

Haplótipos ^1^	Total (N=591)	Angiografia negativa (N=266)	Angiografia positiva (N=335)	p valor
**AAC**	0,241	0,226	0,248	*0,509*
**ATT**	0,205	0,200	0,209	*0,781*
**AAT**	0,150	0,159	0,144	*0,639*
**GAC**	0,140	0,134	0,147	*0,682*
**GAT**	0,120	0,110	0,126	*0,549*
**ATC**	0,095	0,133	0,072	***0,017***
**GTT**	0,024	0,021	0,026	*0,742*
**GTC**	0,025	0,017	0,029	*0,437*

^1^ Teste de Qui-quadrado usado para análise.

A Tabela 4 mostra a diferença entre os genótipos e a atividade da paraoxonase nos grupos de estudo. No total e em ambos os casos e controles, a atividade da paraoxonase estava aumentada na presença do alelo G em comparação com a presença do alelo A. Além disso, a atividade da paraoxonase foi significantemente maior na presença do alelo A de rs850560 em comparação com a presença do alelo T nesse locus e do alelo C de rs705379 no promotor de PON1. As comparações entre os grupos indicaram que a atividade da paraoxonase foi significantemente menor para o genótipo AA do polimorfismo rs662 na DAC em comparação aos controles (p=0,019).

**Tabela 4:** Análise entre grupos de diferentes genótipos de PON1 e atividade de paraoxonase sérica

SNPs		Total (N=591)	p valor^1^	Angiografia negativa (N=266)	p valor^2^	Angiografia positiva (N=335)	p valor^3^	p valor^4^
rs662	*A*	45,45 (27,9-78,75)	*<0,001*	46,80 (28,35-87,75)	*<0,001*	45,00 (26,55-67,50)	*<0,001*	*0,019*
	*G*	94,28 (48,26-148,95)		95,85 (60,19-158,44)		94,28 (48,26(147,15)		*0,527*
	*AA*	40,05 (25,2-57,60)	<0,001*,**,***	41,40 (26,55-58,05)	<0,001*,**,***	40,05 (23,40-57,15)	<0,001*,**,***	*0,345*
	*AG*	81,90 (43,76-126,79)		85,63 (44,55-141,08)		80,10 (43,65-122,85)		*0,084*
	*GG*	129,60 (80,21-175,73)		142,65 (84,60-176,40)		127,35 (62,21-175,28)		*0,933*
**rs854560**	*A*	60,53 (35,51-117,56)	***<0,001***	65,70 (34,65-119,35)	***0,001***	59,40 (36,90-110,59)	***<0,001***	*0,162*
	*T*	41,85 (22,95-79,54)		43,00 (26,55-84,71)		42,08 (22,50-70,88)		*0,585*
	*AA*	67,95 (42,41-129,49)	<0,001*,**,***	80,10 (38,40-130,28)	<0,001*,**	65,48 (43,20(128,81)	<0,001*,**,***	*0,228*
	*AT*	49,50 (28,30-87,75)		57,10 (31,95-95,40)		47,70 (26,55(83,25)		*0,487*
	*TT*	29,25 (17,10-46,80)		30,15 (17,55-43,50)		29,25 (17,10-48,15)		*0,888*
**rs705379**	*C*	65,25 (34,20-122,51)	***<0,001***	78,08 (38,40-133,54)	***0,003***	63,00 (32,85-111,60)	***0,001***	*0,180*
	*T*	47,70 (27,00-87,75)		45,46 (26,55-91,05)		47,70 (29,25-86,85)		*0,530*
	*CC*	75,38 (40,50-144,00)	<0,001*,**,***	83,93 (44,59-156,83)	<0,001*,**	72,90 (36,90-127,35)	<0,001**	*0,329*
	*CT*	54,90 (31,30-98,10)		56,70 (31,46-103,24)		54,90 (31,16-94,40)		*0,299*
	*TT*	41,40 (22,89-65,93)		37,35 (17,55-73,80)		45,45 (24,98-60,30)		*0,770*

Para a análise estatística, teste de Kruskal-Wallis foi usado para comparação entre mais de 2 grupos e teste de Mann-Whitney para comparação entre 2 grupos. ^1^ status de atividade da paraoxonase sérica e SNPs no total. ^2^ status de atividade da paraoxonase sérica e SNPs no grupo angiografia negativa. ^3^ status de atividade da paraoxonase sérica e SNPs no grupo angiografia positiva. ^4^ Comparação entre a atividade da paraoxonase sérica e diferentes genótipos nos grupos angiografia positiva e negativa. *Diferença significante entre homozigotos dominantes e heterozigotos. ** Diferença significante entre dois homozigotos. ***Diferença significante entre homozigotos recessivos e heterozigotos.

## Discussão

Não foi possível demonstrar qualquer associação significante entre os polimorfismos rs850560 e rs705379 e CAD definida angiograficamente em adultos iranianos, enquanto a análise do polimorfismo rs662 mostrou que a homozigosidade para a variante GG *vs.* AA e AG totais estava associada a uma prevalência maior do que 2 vezes na angiografia positiva em comparação com indivíduos com angiografia negativa. Além disso, observamos que a atividade da paraoxonase sérica estava associada a todos os três SNPs avaliados em ambos os grupos de indivíduos. Pode-se dizer que a atividade da enzima paraoxonase era maior nos portadores dos alelos R de rs662, alelo A de rs850560 e alelo C de rs705379. Não houve relação significante entre a positividade da angiografia e a atividade da paraoxonase sérica, embora uma atividade sérica média mais baixa de PON1 tenha sido observada nos pacientes com angiografia positiva.

Em uma metanálise de 17 estudos de diferentes cidades e estados do México realizados em 2018, os genótipos mais associados com atividade enzimática diminuída foram AT/TT de rs850560 e AA de rs662.^22^ Esses resultados foram compatíveis com nossos achados, embora tenha sido uma metanálise nacional conduzida na população do México e, portanto, a etnia era diferente.

Vários estudos investigaram a relação entre os dois SNPs codificadores (rs662 e rs850560) e DAC. A metanálise sugere uma associação entre DAC e PON1. Qinghua Zeng e Juan Zeng sugeriram que o polimorfismo rs662 poderia ser usado para identificar indivíduos altamente suscetíveis a DAC.^23^ Em uma metanálise de 43 estudos que avaliaram 11.000 casos e 13.000 controles. Wheeler et al., mostraram que houve uma relação significante entre o alelo G de rs662 e a DAC, mas não houve associação entre o polimorfismo rs850560 e DAC,^24^ semelhante aos nossos achados. Uma metanálise realizada por Wang et al., de 88 estudos de caso-controle em 2011, relatou resultados semelhantes.^25^

Vaisi-Raygani et al. também encontraram uma relação significante entre o polimorfismo rs662 e DAC no oeste do Irã. Assim, indivíduos com alelo G têm uma chance 1,6 vezes maior de apresentarem DAC.^26^ Por outro lado, Ahmad et al. afirmaram que o alelo G de rs662 está associado ao risco de DAC.^27^ Nossos resultados também são consistentes com outro estudo realizado no norte do Irã, que relatou uma relação entre rs705379 e DAC. Najafi et al. mostraram que, embora esse polimorfismo esteja associado à atividade enzimática sérica, não houve relação significante com a ocorrência de DAC quando comparado ao grupo controle.^28^

Tang et al., avaliaram as atividades séricas da PON-1 e seus determinantes genéticos relacionados em 3.668 indivíduos estáveis (média de idade de 63±11 anos) submetidos à angiografia coronária eletiva (ACE) sem síndrome coronariana aguda e que foram acompanhados prospectivamente para eventos cardiovasculares adversos maiores por um período de 3 anos. Os resultados mostraram que as variantes rs854560 e rs662 tinham fortes efeitos genéticos na atividade sérica de PON1, mas não estavam associadas ao risco de eventos cardíacos adversos maiores (ECAM). Eles sugeriram que os efeitos genéticos desse SNP sobre a atividade da arilesterase são muito fracos para serem observados, especialmente se um limiar biológico mínimo de mudança de atividade for necessário para influenciar o risco de incidentes de ECAM.^29^

Da mesma forma, o estudo GeneBank, um estudo prospectivo com 1.399 indivíduos submetidos à coronariografia diagnóstica eletiva com (idade de 65±11 anos) e sem (idade de 57±12 anos) DAC, mostrou que o polimorfismo rs662 é responsável por cerca de 60% das variações na atividade da PON1. Além disso, a diminuição da atividade sérica de PON1 e seu genótipo AA foram associados a um aumento do estresse oxidativo. Esse genótipo está associado a um aumento na mortalidade e desfechos adversos de eventos cardiovasculares, incluindo infarto do miocárdio e acidente vascular cerebral. Seus resultados mostraram que a incidência desses efeitos adversos foi significantemente menor em indivíduos com maior atividade de PON1.^30^

Um estudo de Ochoa-Martínez et al. observou que os portadores do alelo G do polimorfismo rs662 apresentavam níveis mais altos de biomarcadores de DAC do que os portadores do alelo A.^31^

Liu et al., relataram que o polimorfismo rs662 está significantemente associado à DAC. Em consonância com nossos achados, esse estudo mostrou que os portadores do alelo G desse polimorfismo estão significantemente mais expostos à DAC, e que a atividade e concentração de PON1 estavam positivamente associadas ao alelo G. Além disso, a concentração e a atividade dessa enzima estavam diminuídas nos pacientes com DAC em relação aos controles,^18^ o que não foi encontrado em nosso estudo. Há um paradoxo que já foi explicado por Aviram et al., que relataram que o sítio ativo de PON1 necessário para a proteção contra a oxidação de LDL é diferente daquele necessário para sua atividade da paraoxonase, sugerindo que o alelo R, apesar de apresentar maior atividade em relação ao paraoxon, é defeituoso, pois impede sua atividade antioxidante em relação ao LDL devido ao seu efeito modulador do sítio ativo.^32^ Assim, os portadores do alelo G possuem capacidade reduzida de prevenir a modificação oxidativa de LDL e, consequentemente, são mais suscetíveis a desenvolver DAC do que os portadores do alelo A.^30^

O efeito do SNP rs705379 no promotor do gene PON1 é o outro determinante da atividade de PON1. Brophy et al. mostraram que esse polimorfismo foi responsável por 22,8% da inconsistência na atividade de PON1. Seus resultados mostraram que a atividade de PON1 estava reduzida em 376 indivíduos brancos na presença do alelo T do polimorfismo rS705379 em comparação com a presença do alelo C.^33^ Vários estudos anteriores relataram que o alelo T desse polimorfismo está associado a um risco aumentado de DAC em homens,^14 , 15^ embora não fosse possível confirmar esse resultado, o que pode ser devido a diferenças de idade, etnia, presença de doença, número de participantes inscritos em vários estudos ou diferença no grupo controle. Voetsch et al., mostraram resultados semelhantes em 118 pacientes jovens (idade <45 anos) com um primeiro AVC isquêmico arterial não fatal.^16^ Gupta et al., relataram que a atividade de PON1 está reduzida em pacientes com DAC demonstrada angiograficamente em comparação com indivíduos saudáveis em um grupo étnico discreto de indianos no noroeste do Punjabi, que têm alta incidência de DAC. Além disso, seus resultados mostraram que os polimorfismos rs662 nas regiões codificadoras estão todos independentemente associados à DAC.^34^ James et al. relataram que existe uma associação entre o alelo T de rs705379 e o risco aumentado de DAC em pacientes diabéticos tipo II.^17^

A presença do SNP rs662 por si só não é suficiente para o desenvolvimento de aterosclerose, pois vários estudos anteriores relataram que, além do genótipo, a atividade e as concentrações enzimática também têm papéis importantes.^35^ Além disso, diferenças étnicas,^36^ fatores dietéticos e ambientais,^37^ e o status do HDL^38^ podem afetar o fenótipo de PON1. Além disso, um estudo destacou a importância do HDL, que é um fator relacionado à DAC, e que envolve PON1.^39^ Foi relatado que os três genótipos AA, AG e GG apresentam valores médios semelhantes de níveis de PON1/HDL. Este resultado pode explicar por que tentativas anteriores de correlacionar o genótipo rs662 com uma predisposição para aterosclerose falharam e abre caminho para novas metodologias de fenotipagem de PON1 que podem fornecer uma melhor correlação.^40^ Uma limitação do nosso estudo foi o uso de indivíduos com estenose <30% como controles. Idealmente, os controles seriam indivíduos com DAC mínima definida angiograficamente, mas esses indivíduos raramente necessitam de angiografia nessa idade.

## Conclusões

Descobrimos que portadores do alelo G do polimorfismo Q192R do gene PON1 estavam independentemente associados a uma angiografia coronária positiva. Além disso, portadores do alelo G de rs662, alelo A de rs850560 e alelo C de rs705379 têm níveis aumentados de atividade sérica de PON1. Não foi possível estabelecer qualquer relação significante entre a atividade da paraoxonase sérica e uma angiografia positiva em uma amostra do nordeste do Irã.

## *Material suplementar

Para informação adicional, por favor, clique aqui


