# Supplementary material for: Associação do Genótipo e Fenótipo da Paraoxonase-1 com Angiografia Positiva para Doença Arterial Coronariana
Source: Arq Bras Cardiol. 2022 Aug 24;119(4):593–601. [Article in Portuguese] doi: 10.36660/abc.20210422 (PMC9563872; doi:10.36660/abc.20210422)
Supplement: Supplementary file 1 [file 2021-0422-Suplemento_1.pdf]

Suplemento 1- Primers e protocolos de PCR

SNP		Primers	Protocolo de PCR		
			Ciclo	Temp. (°C)	Tempo
rs662	Alelo A *Forward*	5´-CTATTTTCTTGACCCCTACTTTCA-3´	1	95	10 min
			35	95	30s
	Alelo G *Forward*	5´-ACTATTTTCTTGACCCCTACTTATG-3´		58	30s
				72	30s
	Reverse	5´-AGTTCACATACTTGCCATCGG-3´	1	72	10 min
rs854560	Alelo A *Forward*	5´-GTCCATTAGGCAGTATCTCCGA-3´	1	95	10 min
			35	95	30s
	Alelo T *Forward*	5´-AGTCCATTAGGCAGTATCTCCGT-3´		62	30s
				72	30s
	Reverse	5´-CCCAGTTTCAAGTGAGGTGTGA-3´	1	72	10 min

rs705379	*Forward*	5´-TGCAGCCGCAGCCCTGCTGGGGCAGCGCCGATTGGCCCGCCGC-3´	1	95	10 min
			35	95	30s
	Reverse	5´-GACCGCAAGCCACGCCCTCTGTGCACC-3'		72	30s
			1	72	10min
