# Supplementary material for: Associação do Genótipo e Fenótipo da Paraoxonase-1 com Angiografia Positiva para Doença Arterial Coronariana
Source: Arq Bras Cardiol. 2022 Aug 24;119(4):593–601. [Article in Portuguese] doi: 10.36660/abc.20210422 (PMC9563872; doi:10.36660/abc.20210422)
Supplement: Supplementary file 2 [file 2021-0422-Supplement_1.pdf]

Supplement 1- Primers and PCR protocols

SNP		Primers	PCR protocol		
			Cycle	Temp. (° C)	Time
rs662	Forward Allele A	5´-CTATTTTCTTGACCCCTACTTTCA-3´	1	95	10 min
			35	95	30s
	Forward Allele G	5´-ACTATTTTCTTGACCCCTACTTATG-3´		58	30s
				72	30s
	Reverse	5´-AGTTCACATACTTGCCATCGG-3´	1	72	10 min
rs854560	Forward Allele A	5´-GTCCATTAGGCAGTATCTCCGA-3´	1	95	10 min
			35	95	30s
	Forward Allele T	5´-AGTCCATTAGGCAGTATCTCCGT-3´		62	30s
				72	30s
	Reverse	5´-CCCAGTTTCAAGTGAGGTGTGA-3´			

			1	72	10 min
rs705379	Forward	5́-TGCAGCCGCAGCCCTGCTGGGGCAGCGCCGATTGGCCCGCCGC-3́	1	95	10 min
			35	95	30s
	Reverse	5́-GACCGCAAGCCACGCCCTCTGTGCACC-3'		72	30s
			1	72	10min
