# Supplementary material for: Associação do Genótipo e Fenótipo da Paraoxonase-1 com Angiografia Positiva para Doença Arterial Coronariana
Source: Arq Bras Cardiol. 2022 Aug 24;119(4):593–601. [Article in Portuguese] doi: 10.36660/abc.20210422 (PMC9563872; doi:10.36660/abc.20210422)
Supplement: Translation

# Translation: Association of Paraoxonase-1 Genotype and Phenotype with Angiogram Positive Coronary Artery Disease

**Authors:** Sara Saffar Soflaei, Mojtaba Baktashian, Kiana Hosseinpour Moghaddam, Maryam Saberi-Karimian, Negin Kosari, Seyed Mohammad Hashemi, Mohsen Mouhebati, Mahsa Amini, Mashallah Dehghani, Habibollah Esmaily, Mahmoud Ebrahimi, Homa Falsoleiman, Abolfazl Nosrati-Tirkani, Fatemeh Sadabadi, Gordon A. Ferns, Mansoor Salehi, Alireza Pasdar, Majid Ghayour-Mobarhan

**Affiliations:** 1 Iran International UNESCO Center for Health-Related Basic Sciences and Human Nutrition, Mashhad University of Medical Sciences, Mashhad – Iran; 2 Iran Department of Biology, Faculty of Sciences, Ferdowsi University of Mashhad, Mashhad – Iran; 3 Iran Metabolic Syndrome Research Center, Faculty of Medicine, Mashhad University of Medical Sciences,Mashhad – Iran; 4 Iran Department of Cardiology, Chamran Hospital, School of Medicine, Isfahan University of Medical Sciences,Isfahan – Iran; 5 Iran Cardiovascular Research Center, School of Medicine, Mashhad University of Medical Sciences, Mashhad – Iran; 6 Iran Department of Biostatistics and Epidemiology, School of Health, Mashhad University of Medical Sciences,Mashhad – Iran; 7 Iran Biochemistry of Nutrition Research Center, Faculty of Medicine, Mashhad University of Medical Sciences, Mashhad – Iran; 8 UK Brighton & Sussex Medical School, Division of Medical Education, Falmer, Brighton – UK; 9 Iran Department of genetics, Faculty of medicine and genetics laboratory AL Zahra hospital, Isfahan University of Medicine, Isfahan – Iran; 10 UK Division of Applied Medicine, Medical School, University of Aberdeen, Foresterhill, Aberdeen – UK

**Keywords:** Coronary Artery Disease, Angiography, Aryldialkylphosphatase

## Abstract

**Background:**

It has been shown that increased serum PON1 levels are protective against several disorders. Several single nucleotide polymorphisms (SNPs) of the PON1 gene have been reported to be associated with serum enzyme protein levels and activity.

**Objective:**

To investigate the association of SNPs of PON1 and serum paraoxonase activity with coronary artery disease (CAD).

**Methods:**

A total of 601 unrelated patients who underwent coronary angiography including those who had >50% stenosis (N=266) and those with <30% stenosis (N=335) were studied. The Paraoxonase gene rs662 and rs840560 SNPs were determined using the ARMS-PCR method and the rs705379 SNP was genotyped using PCR-RFLP analysis. Serum paraoxonase activity was measured using paraoxon as a substrate. A p value of p<0.05 was considered as significant.

**Results:**

Serum paraoxonase activity was not significantly different between the study groups. After adjustment for age, sex, hypertension, diabetes mellitus and dyslipidemia, the GG genotype and co-dominant model of rs662 was positively associated with a positive angiogram (respectively, OR=2.424, 95%CI [1.123-5.233], p<0.05, OR=1.663, 95%CI [1.086-2.547]). Serum paraoxonase activity was significantly higher in the G allele and GG variant of rs662, A allele and AA variant of rs854560 and C allele and CC variant of rs705379. The haplotype analysis has shown that the ATC haplotype was significantly more prevalent among the angiogram negative group. The analysis between groups indicated that the A allele of rs662 was significantly associated with lower paraoxonase activity in the positive angiogram group (p=0.019).

**Conclusions:**

The presence of the G allele of the rs662 single nucleotide polymorphism is independently associated to increased risk of CAD.

## Introduction

The World Health Organization (WHO) reports that 71% of deaths each year are due to non-communicable diseases, of which 43% are due to coronary artery disease (CAD).^1^This was also reported to be approximately 46% in Iran in 2019,^2^where the prevalence of CAD is increasing.^3 , 4^

Oxidative stress has a key role in the initiation and progression of atherosclerosis,^5^as well as in the pathogenesis of CAD and its related outcomes.^6^It has been shown that high density lipoprotein (HDL) has antioxidant and anti-inflammatory properties, in which paraoxonase 1 (PON1) may play a role by decreasing the production of oxidized low density lipoprotein (LDL) during the process of lipid peroxidation. PON1 is a calcium-dependent esterase, of which serum concentration differs by ethnicity and geographically. It has been shown that a decreased PON1 activity is associated with conditions in which there is oxidative stress, including metabolic syndrome, CAD, Alzheimer and aging, and in this case, increased PON1 levels may be protective.^7^The paraoxonase gene cluster encodes for three distinct members: PON1-PON2 and PON3, which are located on chromosome 7q21.3. More than 160 single nucleotide polymorphisms (SNPs) have been identified for the PON1 gene,^8^of which the rs662, rs854560, rs705379 are reported to be associated with serum enzyme protein levels and activity. The rs662 and rs850560 SNPs are located within coding regions and exert an amino acid substitution,^9 , 10^whereas the third polymorphism, rs705379, is located on the promoter region.^11^The presence of rs662 results in a glutamine-to-arginine substitution, increases the rate of hydrolysis of paraoxon and chlorpyrifos-oxon. While the rs850560 polymorphism, which results in a leucine-to-methionine amino acid substitution, is also associated with decreased serum PON1.^12^The rs705379 SNP occurs at the binding site of the transcription factor Sp1, and has the greatest effect on the expression of PON1.^11^This polymorphism accounts for approximately 30% of variations in plasma PON1 levels. The C allele of rs705379 is associated with an increased promoter activity, and therefore the expression of the PON1 gene is augmented.^13^Moreover, several studies have shown that this polymorphism is associated with an increased risk of CAD, especially in young people^14 - 16^and in individuals with type 2 diabetes.^17^

Since CAD is an important disease in relation to mortality and studies have shown that there is an association between PON1 and CAD, it was decided to investigate this association in the Iranian society, especially in the northeast of the country.^18^There are few studies on the association between PON1 genotype, or phenotype and CAD in northeastern Iran^19 - 21^and serum enzyme activity was not studied along with the polymorphisms. The aim of current study was to assess the association between PON1 polymorphisms and paraoxonase activity with CAD among Iranian adults living in northeastern Iran.

## Material and methods

### Study design and population

This case-control study was carried out between December 2014 and April 2017; 601 unrelated Iranian patients who underwent elective coronary angiography were recruited. Patients were referred for angiography because of chest pain or equivalent symptoms, such as dyspnea on exertion. Based on the results of the angiography, the patients were divided into two groups: those with obstructive coronary artery disease with coronary stenosis >50% in at least one coronary artery (N=266) (angiogram positive) and patients with non-obstructive coronary artery disease with stenosis <30% in coronary arteries (N=335) (angiogram negative).

Demographic data including sex, age, smoking history, past history of diabetes mellitus (DM), hypertension (HTN), and dyslipidemia were collected from the medical records. Systolic blood pressure (SBP) and diastolic blood pressure (DBP) were measured right before the procedure. Patients with autoimmune disorders, active cancer, thrombophilia or chronic kidney disease were excluded.

Blood samples were taken before the procedure into EDTA tubes for DNA extraction and into tubes with no anticoagulant for paraoxonase activity measurement. Serum was separated by centrifuging the blood for 15 min at 1000 rpm speed (manufacturer’s recommended speed) (BEHDAD, Iran) and stored at -80°C.

### Genotyping

DNA was extracted from EDTA blood, using a genomic DNA isolation kit (Genet bio, Korea) based on the manufacturer’s instructions. DNA Purity and quantification were determined by UV spectrophotometry (Infinite 200PRONanoQuant, Tecan).

Three SNPs of PON1 genes were genotyped. We used the double ARMS-PCR method for the rs662 and rs854560 SNPs and the PCR-RFLP method for rs705379 SNP. Details on the primers used and PCR conditions are detailed in Supplement 1. Gel electrophoresis was performed using 2% agarose in TBE for the three SNPs. To determine the genotypes of rs705379, we used 5 units of Bsh1236I (Thermo Scientific) for 16 hours at 37°C. The 109bp PCR product was cut into 67bp and 42bp fragments and visualized with UV-Trans-illuminator. Sequencing was performed to confirm the accuracy of the genotyping techniques.

### Paraoxonase activity

Paraoxonase activity was measured by adding 10μL of serum to 290 μL of Tris-HCl buffer (100mmol/L, pH=8.0) containing 1mmol/L CaCl_2_and 1mmol/L paraoxon (D9286, Sigma Chemical Company, Deisenhofen, Germany). The generation of p-nitrophenol was measured at 405 nm and at room temperature using a plate reader (EPOCH, USA) 3 and 6 minutes after adding paraoxon as the substrate. Paraoxonase activity was reported in Units per liter of serum per minute.

### Ethical considerations

All participants filled out a written informed consent form. The Ethics Committee at Mashhad University of Medical Sciences approved the study protocol (ID Number: 930834).

### Statistical analysis

All data were statistically analyzed using the Statistical Package for Social Sciences software (SPSS Inc., IL, USA). Data normality was checked by the Kolmogorov-Smirnov test. Normally distributed variables were expressed in Mean ± standard deviation (SD) and variables without a normal distribution were described using median and interquartile range. Categorical variables were reported by number and percentage. For the analysis between groups, the chi-square test was used for categorical data, and the independent sample t-test for quantitative data (for normally distributed data) or Mann-Whitney and Kruskal-Wallis (for non-normally distributed data), respectively. Univariate and multivariate analyses with binary logistic regression were performed to indicate the association between SNPs and positive angiography, being expressed in OR (95%CI). Statistical significance was set at p <0.05. Haplotype analysis was performed using SNPAlyze (demo version, V8.1.1).

## Results

Differences in baseline characteristics between the study groups are shown in table 1 . The differences between the frequencies of the three genotypes and angiogram positivity are shown in Table 2 . After adjusting for age, sex, HTN, DM and dyslipidemia, a recessive model for GG genotype of rs662 was significant between the study populations. Also, a significant result was observed in the co-dominant model for rs662.

**Table 1:** Baseline characteristics of the study population

Variable		Angiogram negative (N=266)	Angiogram positive (N=335)	p Value
Age (y) (Mean±SD)^1^		55.70±10.96	61.53±8.91	<0.001
Sex (N%)^2^	*Male*	130 (48.9%)	227 (69.8%)	<0.001
	*Female*	136 (51.1%)	98 (30.2%)	
**Positive smoking history (N %)^2^**		42 (16.0%)	52 (16.5%)	0.890
**HTN history (N %)^2^**		121 (45.5%)	192 (59.3%)	0.001
**DM history (N %)^2^**		70 (26.3%)	130 (40.5%)	<0.001
**Dyslipidemia history (N %)^2^**		93 (35.1%)	178 (55.3%)	<0.001
**BMI (N %)^2^**	*Normal (BMI<25)*	87 (37.5%)	104 (35.6%)	0.683
	*Overweight (25≤BMI<30)*	101 (43.5%)	138 (47.3%)	
	*Obese (BMI≥30)*	44 (19.0%)	50 (17.1%)	
**SBP (Mean ± SD)^1^**		121.81±17.37	124.93±15.44	0.035
**DBP (Mean ± SD)^1^**		76.12±10.42	77.75±8.75	0.063
**Serum Paraoxonase activity (U/L) (Median(IQR))^1^**		57.60(32.70-105.15)	52.20(30.38-95.18)	0.237

HTN: hypertension; DM: diabetes mellitus; BMI: body mass index; SBP: systolic blood pressure; DBP: diastolic blood pressure.^1^Analysis by t-test or Mann-Whitney test, when necessary.^2^Analysis by Chi-square test.

**Table 2:** Association between PON1 polymorphisms and CAD

Genotypes			Angiogram negative (N=266)	Angiogram positive (N=335)	Univariate regression	Multivariate regression^1^
rs662	*A*		0.70	0.68	1.093 (0.847-1.410)	1.062 (0.773-1.460)
	*G*		0.30	0.32		
	*AA*		112(44.8%)	150(48.1%)	Ref.	Ref.
	*AG*		121(48.4%)	121(38.8%)	0.747 (0.525-1.061)	0.685 (0.439-1.069)
	*GG*		17(6.8%)	41(13.1%)	1.913 (1.022-3.583)*	1.802 (0.827-3.925)
	*Dominant model*	*AA*	112(44.8%)	150(57.3%)	0.833 (0.632-1.233)	0.818 (0.535-1.250)
		*AG+GG*	138(55.2%)	162(51.9%)		
	*Recessive model*	*AA+AG*	234(93.6%)	271(86.9%)	2.360 (1.274-4.373)**	2.424 (1.123-5.233)*
		*GG*	16(6.4%)	41(13.1%)		
	*Additive model*	*AA*	112(87.5%)	150(78.5%)	2.041 (1.076-3.871)*	2.080 (0.900-4.810)
		*GG*	16(12.5%)	41(21.5%)		
	*Co-dominant model*	*AG*	122(48.8%)	121(38.9%)	1.508 (1.077-2.113)*	1.663 (1.086-2.547)*
		*AA+GG*	128(51.2%)	190(61.1%)		
**rs854560**	*A*		0.62	0.67	0.944 (0.682-1.309)	0.836 (0.583-1.200)
	*T*		0.38	0.33		
	*AA*		88(35.5%)	131(42.3%)	Ref.	Ref.
	*AT*		131(52.8%)	149(48.1%)	1.053 (0.692-1.603)	0.918 (0.550-1.533)
	*TT*		29(11.7%)	30(9.7%)	0.299(0.694-1.383)	0.603(0.260-1.399)
	*Dominant model*	*AA*	86(34.7%)	130(41.9%)	1.004(0.643-1.568)	0.854(0.523-1.393)
		*AT+TT*	162(65.3%)	180(58.1%)		
	*Recessive model*	*AA+AT*	220(88.7%)	279(90.0%)	0.770(0.381-1.556)	0.631(0.285-1.398)
		*TT*	28(11.3%)	31(10.0%)		
	*Additive model*	*AA*	86(75.4%)	130(81.2%)	0.789(0.374-1.665)	0.601(0.259-1.396)
		*TT*	28(24.6%)	30(18.8%)		
	*Co-dominant model*	*AT*	134(54.0%)	149(48.2%)	0.909(0.586-1.412)	0.990 (0.610-1.607)
		*AA+TT*	114(46.0%)	160(51.8%)		
**rs705379**	*C*		0.50	0.49	1.055 (0.798-1.394)	0.941(0.669-1.322)
	*T*		0.50	0.51		
	*CC*		49(24.1%)	68(21.9%)	Ref.	Ref.
	*CT*		109(53.7%)	171(55.0%)	0.941(0.574-1.544)	1.079(0.589-1.975)
	*TT*		45(22.2%)	72(23.2%)	1.098 (0.603-2.000)	0.585(0.418-1.761)
	*Dominant model*	*CC*	52 (25.6%)	67(21.4%)	1.086 (0.642-1.838)	1.143 (0.645-2.028)
		*CT+TT*	151(74.4%)	246(78.6%)		
	*Recessive model*	*CC+CT*	159(78.3%)	242(77.3%)	1.001 (0.600-1.703)	0.820 0.460-1.462)
		*TT*	44(21.7%)	71(22.7%)		
	*Additive model*	*CC*	52(54.2%)	67(48.6%)	1.075 (0.561-2.063)	0.939 (0.458-1.924)
		*TT*	44(45.8%)	71(51.4%)		
	*Co-dominant model*	*CT*	107(52.7%)	175(55.9%)	0.952 (0.613-1.478)	0.791 (0.486-1.288)
		*CC+TT*	96(47.3%)	138(44.1%)		

Analysis was performed using univariate and multivariate regression. *P value ≤ 0.05. **P value ≤ 0.01.^1^Adjusted for age, sex, hypertension, diabetes mellitus, dyslipidemia.

The haplotype analysis showed that the “ATC” haplotype showed a significant difference between the two analyzed groups (p=0.017) ( Table3 ).

**Table 3:** Difference between haplotype frequencies in the study groups

Haplotypes^1^	Total (N=591)	Angiogram negative (N=266)	Angiogram positive (N=335)	p value
**AAC**	0,241	0,226	0,248	*0,509*
**ATT**	0,205	0,200	0,209	*0,781*
**AAT**	0,150	0,159	0,144	*0,639*
**GAC**	0,140	0,134	0,147	*0,682*
**GAT**	0,120	0,110	0,126	*0,549*
**ATC**	0,095	0,133	0,072	***0,017***
**GTT**	0,024	0,021	0,026	*0,742*
**GTC**	0,025	0,017	0,029	*0,437*

^1^Chi-square test used for the analysis.

Table 4 shows the difference between genotypes and paraoxonase activity in the study groups. In total and in both cases and controls, paraoxonase activity was increased in the presence of the G allele in comparison with the presence of the A allele. Moreover, paraoxonase activity was significantly higher in the presence of the A allele of rs850560 in comparison with the presence of the T allele at this locus and the C allele of rs705379 in PON1 promoter. Comparisons between the groups indicated that paraoxonase activity was significantly lower for the AA genotype of the rs662 polymorphism in CAD when compared to the controls (p=0.019).

**Table 4:** Analysis between groups of different PON1 genotypes and serum paraoxonase activity

SNPs		Total (N=591)	p value^1^	Angiogram negative (N=266)	p value^2^	Angiogram positive (N=335)	p value^3^	p value^4^
rs662	*A*	45.45 (27.9-78.75)	*<0.001*	46.80 (28.35-87.75)	*<0.001*	45.00 (26.55-67.50)	*<0.001*	*0.019*
	*G*	94.28 (48.26-148.95)		95.85 (60.19-158.44)		94.28 (48.26(147.15)		*0.527*
	*AA*	40.05 (25.2-57.60)	<0.001*,**,***	41.40 (26.55-58.05)	<0.001*,**,***	40.05 (23.40-57.15)	<0.001*,**,***	*0.345*
	*AG*	81.90 (43.76-126.79)		85.63 (44.55-141.08)		80.10 (43.65-122.85)		*0.084*
	*GG*	129.60 (80.21-175.73)		142.65 (84.60-176.40)		127.35 (62.21-175.28)		*0.933*
**rs854560**	*A*	60.53 (35.51-117.56)	***<0.001***	65.70 (34.65-119.35)	***0.001***	59.40 (36.90-110.59)	***<0.001***	*0.162*
	*T*	41.85 (22.95-79.54)		43.00 (26.55-84.71)		42.08 (22.50-70.88)		*0.585*
	*AA*	67.95 (42.41-129.49)	<0.001*,**,***	80.10 (38.40-130.28)	<0.001*,**	65.48 (43.20(128.81)	<0.001*,**,***	*0.228*
	*AT*	49.50 (28.30-87.75)		57.10 (31.95-95.40)		47.70 (26.55(83.25)		*0.487*
	*TT*	29.25 (17.10-46.80)		30.15 (17.55-43.50)		29.25 (17.10-48.15)		*0.888*
**rs705379**	*C*	65.25 (34.20-122.51)	***<0,001***	78.08 (38.40-133.54)	***0,003***	63.00 (32.85-111.60)	***0.001***	*0.180*
	*T*	47.70 (27.00-87.75)		45.46 (26.55-91.05)		47.70 (29.25-86.85)		*0.530*
	*CC*	75.38 (40.50-144.00)	<0.001*,**,***	83.93 (44.59-156.83)	<0.001*,**	72.90 (36.90-127.35)	<0.001**	*0.329*
	*CT*	54.90 (31.30-98.10)		56.70 (31.46-103.24)		54.90 (31.16-94.40)		*0.299*
	*TT*	41.40 (22.89-65.93)		37.35 (17.55-73.80)		45.45 (24.98-60.30)		*0.770*

For statistical analysis, Kruskal–Wallis test was used for comparison between more than 2 groups and Mann-Whitney test for comparison between 2 groups.^1^serum paraoxonase activity status and SNPs in total.^2^serum paraoxonase activity status and SNPs in the angiogram negative group.^3^serum paraoxonase activity status and SNPs in the angiogram positive group.^4^Comparison between serum paraoxonase activity and different genotypes in angiogram positive and negative groups. *Significant difference between dominant homozygous and heterozygous. **Significant difference between two homozygous. ***Significant difference between recessive homozygous and heterozygous.

## Discussion

We could not show any significant association between rs850560 and rs705379 polymorphisms and angiographically defined CAD in Iranian adults, whereas the analysis of the rs662 polymorphism showed that homozygosity for the GG variant vs. total AA and AG was associated with a more than a 2-fold higher prevalence in the positive angiogram when compared to the negative angiogram subjects. Moreover, we found that serum paraoxonase activity was associated with all three assessed SNPs in both groups of subjects. It can be said that the paraoxonase enzyme activity was higher in carriers of R alleles of rs662, A allele of rs850560 and C Allele of rs705379. There was no significant relationship between angiogram positivity and serum paraoxonase activity, although a lower mean serum PON1 activity was observed in the angiogram positive patients.

In a meta-analysis on 17 studies carried out in different cities and states of Mexico conducted in 2018, the most frequently associated genotypes with decreased enzyme activity were AT/TT of rs850560 and AA of rs662.^22^These results were compatible with our findings, although this was a national meta-analysis conducted in the Mexico population and, therefore, the ethnicity was different.

Several studies have investigated the relationship between the two coding SNPs (rs662 and rs850560) and CAD. The meta-analysis suggests an association between CAD and PON1. Qinghua Zeng and Juan Zeng suggested that the rs662 polymorphism could be used to identify individuals that are highly susceptible to CAD.^23^In a meta-analysis on 43 studies that assessed 11,000 cases and 13,000 controls, Wheeler et al. showed there was a significant relationship between the G allele of the rs662 polymorphism and CAD, but there was no association between the rs850560 polymorphism and CAD,^24^similar to our findings. A meta-analysis conducted by Wang et al., of 88 case-control studies in 2011 reported similar results.^25^

Vaisi-Raygani et al., also found a significant relationship between the rs662 polymorphism and CAD in western Iran. Thus, individuals with the G allele have a 1.6-fold chance of having CAD.^26^On the other hand, Ahmad et al. stated that the G allele of rs662 is associated with the risk of developing CAD.^27^Our results are also consistent with another study carried out in the northern Iran, which reported that there was a relationship between rs705379 and CAD. Najafi et al. showed that, while this polymorphism is associated with serum enzyme activity, there was no significant relationship with the occurrence of CAD when compared to the control group.^28^

Tang et al., evaluated serum PON-1 activity and its related genetic determinants in 3,668 stable subjects (mean age of 63±11) undergoing elective coronary angiography (ECA) without acute coronary syndrome and were prospectively followed for major adverse cardiovascular events for a period of 3 years. Their results showed that the rs854560 and rs662 variants had strong genetic effects on serum PON1 activity but were not associated with the risk of major adverse cardiac events (MACE). They suggested that the genetic effects of this SNP on arylesterase activity are too weak to be observed, especially if a minimum biological threshold of activity change is needed to influence the risk of MACE incidents.^29^

Similarly, the GeneBank study, a prospective study on 1,399 individuals undergoing elective diagnostic coronary angiography with (aged 65±11 years) and without (aged 57±12 years) CAD, showed that the rs662 polymorphism accounts for about 60% of the variations in PON1 activity. Moreover, decreased serum activity of PON1 and its AA genotype were associated with an increase in oxidative stress. This genotype is associated with an increase in mortality and adverse outcomes of cardiovascular events, including myocardial infarction and stroke. Their results showed that the incidence of these adverse effects was significantly lower in individuals with higher PON1 activity.^30^

A study by Ochoa-Martínez et al. found that carriers of the G allele of the rs662 polymorphism had higher levels of CAD biomarkers than carriers of the A allele.^31^

Liu et al., have reported that the rs662 polymorphism is significantly associated with CAD. In line with our findings, this study showed that the carriers of this polymorphism G allele are significantly more exposed to CAD and the activity and concentration of PON1 have been positively associated with the G allele. In addition, the concentration and activity of this enzyme was decreased in patients with CAD compared to the controls,^18^which was not found in our study. There is a paradox, which was previously explained by Aviram et al., who reported that the PON1 active site required for protection against LDL oxidation is different from the one required for its paraoxonase activity, suggesting that the R allele, despite showing higher activity toward paraoxon, is defective as it prevents its antioxidant activity toward LDL due to its active site modulating effect.^32^Thus, carriers of the G allele have reduced capacity to prevent the oxidative modification of LDL and, consequently, are more susceptible to develop CAD than carriers of the A allele.^30^

The rs705379 SNP effect on the PON1 gene promoter is the other determinant of PON1 activity. Brophy et al., have shown that this polymorphism accounted for 22.8% of the inconsistency in PON1 activity. Their results showed that PON1 activity was decreased in 376 white individuals in the presence of the T allele of the rS705379 polymorphism in comparison with presence of the C allele.^33^Several previous studies have reported that the T allele of this polymorphism is associated with an increased risk of CAD in males,^14 , 15^although we could not confirm this result, which may be due to differences in age, ethnicity, presence of disease, the number of enrolled participants in various studies or difference in control group. Voetsch et al., have shown similar results in a group of 118 young patients (aged <45 years) with a first nonfatal arterial ischemic stroke.^16^Gupta et al. have reported that PON1 activity is reduced in angiographically CAD patients when compared to healthy subjects in a discretely ethnic Indian group in northwest Punjabi who have high incidence of CAD. Moreover, their results showed that the rs662 polymorphisms in the coding regions are all independently associated with CAD.^34^James et al. have reported that there is an association between the T allele of rs705379 and increased risk of CAD in type II diabetic patien.^17^

The presence of the rs662 SNP alone is not enough for atherosclerosis development, because several previous studies have reported that in addition to genotype, enzyme activity and concentrations have important roles, too.^35^Moreover, ethnic differences,^36^dietary and environmental factors,^37^and HDL status^38^can all have an effect on the PON1 phenotype. In addition, a study highlighted the importance of HDL, which is a CAD-related factor, in that PON1 is involved.^39^It has been reported that all three genotypes AA, AG, and GG result in similar mean values of PON1/HDL levels. This result may explain why previous attempts to correlate the rs662 genotype with a predisposition for atherosclerosis failed and opens the road for new PON1 phenotyping methodologies that may provide a better correlation.^40^One limitation of our study was the use of subjects with <30% stenosis as controls. Ideally, the controls would be individuals with minimal angiographically-defined CAD, but these individuals uncommonly require angiography at this age.

## Conclusions

We have found that carriers of the G allele of the Q192R polymorphism of the PON1 gene were independently associated with a positive coronary angiogram. Moreover, carriers of G allele rs662, A allele of rs850560 and C allele of rs705379 have increased levels of serum PON1 activity. We could not establish any significant relationship between serum paraoxonase activity and a positive angiogram in a sample from northeastern Iran.

## * Supplemental Materials

For additional information, please, click here
